# Anxiety Shapes Amygdala-Prefrontal Dynamics During Movie Watching

**DOI:** 10.1016/j.bpsgos.2022.03.009

**Published:** 2022-03-31

**Authors:** Peter A. Kirk, Avram J. Holmes, Oliver J. Robinson

**Affiliations:** a Institute of Cognitive Neuroscience, University College London, London, United Kingdom; b Experimental Psychology, University College London, London, United Kingdom; c Clinical, Educational and Health Psychology, University College London, London, United Kingdom; d Departments of Psychology and Psychiatry, Yale University, New Haven, Connecticut; e Wu Tsai Institute, Yale University, New Haven, Connecticut

**Keywords:** Amygdala, Anxiety, fMRI, Naturalistic, Vigilance

## Abstract

**Background:**

A well-characterized amygdala–dorsomedial prefrontal circuit is thought to be crucial for threat vigilance during anxiety. However, engagement of this circuitry within relatively naturalistic paradigms remains unresolved.

**Methods:**

Using an open functional magnetic resonance imaging dataset (Cambridge Centre for Ageing Neuroscience; *n* = 630), we sought to investigate whether anxiety correlates with dynamic connectivity between the amygdala and dorsomedial prefrontal cortex during movie watching.

**Results:**

Using an intersubject representational similarity approach, we saw no effect of anxiety when comparing pairwise similarities of dynamic connectivity across the entire movie. However, preregistered analyses demonstrated a relationship between anxiety, amygdala-prefrontal dynamics, and anxiogenic features of the movie (canonical suspense ratings). Our results indicated that amygdala-prefrontal circuitry was modulated by suspense in low-anxiety individuals but was less sensitive to suspense in high-anxiety individuals. We suggest that this could also be related to slowed habituation or amplified anticipation. Moreover, a measure of threat-relevant attentional bias (accuracy/reaction time to fearful faces) demonstrated an association with connectivity and suspense.

**Conclusions:**

Overall, this study demonstrated the presence of anxiety-relevant differences in connectivity during movie watching, varying with anxiogenic features of the movie. Mechanistically, exactly how and when these differences arise remains an opportunity for future research.

Studies to date have outlined an amygdala-prefrontal circuit (dorsomedial/anterior cingulate) that may underlie threat processing (1,2). Conditioning paradigms have demonstrated this in vitro in both rodents (3) and primates (4). Functional magnetic resonance imaging (fMRI) studies have provided evidence for the presence of this circuit in humans (2,5). Moreover, the degree of engagement appears to interact with the affective content of stimuli (e.g., facial expressions) and individual differences in trait anxiety (2,5). As such, recruitment of this circuit, above and beyond regional activation (6, 7, 8), is thought to drive attentional amplification of threat-relevant features in the environment, a core component of anxiety (9). However, studies to date have primarily investigated this using static stimuli (i.e., faces) presented without context. Consequently, the relationship between this circuit and anxiety in more dynamic, naturalistic contexts remains poorly understood. Extending study of this circuitry to more naturalistic stimuli offers the opportunity to validate these findings in more ecologically rich settings and observe how this circuit may be modulated as a function of dynamic contextual features.

A small number of studies have demonstrated anxiety-relevant within-subject amygdala-prefrontal coupling during movie watching. Specifically, these have demonstrated increased functional connectivity between the amygdala and dorsomedial prefrontal cortex (dmPFC) during fear-inducing/anxiogenic scenes within movies (10,11). However, we know little about how individual differences in anxiety interact with this connectivity during movie watching. Therefore, we previously explored how between-subject differences in anxiety modulated this circuitry. We did not find convincing interactions between circuitry and individual differences in anxiety using traditional, static (time-invariant) feature- and seed-based approaches (12). Put more simply, when looking for connectivity patterns that were stable (irrespective of specific scenes within movies), we did not see differences as a function of anxiety. Given the emotional complexity of movies, an approach that is sensitive to ongoing dynamics (e.g., how anxiety-inducing a scene is) within the stimuli may be more suitable.

Studies have now started to implement intersubject representational similarity analysis for movie stimuli [for an introduction to representational similarity analysis, see (13)]. Broadly speaking, this treats other subjects as a control measure for each subject at every point in the movie. By comparing two subjects’ brain activity across a movie, we can generate a measure of how neurally similar the two subjects were. If differences in neural similarity correlate with differences in self-reported similarity (i.e., trait anxiety), we can infer that brain activity in region X during movie watching varies as a function of individual difference Y. This technique is unconstrained in that it does not rely on traditional onset-convolved regressors, which require the researcher to specify exact events. However, unlike stimulus-independent analyses, such as seed-based resting-state analysis, it remains stimulus driven and time locked: the movie elicits a generally shared experience, but similarity measures will capture, in this instance, anxiety-relevant deviations from this. This approach has demonstrated sensitivity to detecting shared and idiosyncratic representations relevant to affective systems (14, 15, 16, 17). With a goal of predicting individual differences (i.e., self-report scores), it has been argued that this approach allows for greater sensitivity for detecting brain-behavior relationships versus traditional resting-state paradigms (14). However, despite being sensitive to the content within a movie, results do not allow for temporal specificity (which time points in the movie are driving effects) because similarity measures are based on comparisons across the entirety of movie-viewing. Thus, this approach may offer sensitivity for detecting phenotypic variation and clustering, but its ability to inform biopsychological theories of affective systems is inherently limited.

A complementary method to deriving similarity measures across an entire movie is through dynamic (time-varying) analyses. This provides information regarding neural connectivity at each time point, allowing brain measures to be mapped back onto stimulus information (e.g., anxiogenic features). This works in a similar manner to the aforementioned analyses (looking at similarity across the entire movie), except neural similarity is based on specific time points throughout the movie. Similar to traditional techniques (feature-based regression), this allows for inferences concerning which time points are driving effects. However, unlike traditional modeling approaches, this also makes fewer assumptions regarding properties of the fMRI signal, such as shape of hemodynamic response function across anatomy and time. Consequently, this has the potential to increase sensitivity while retaining stimulus-relevant specificity. Little work has been done in this domain; yet, this approach has demonstrated that the relationship between depressive symptoms and brain activity (i.e., medial PFC and posterior cingulate) tracks ongoing emotional intensity/valence of movie stimuli (18). It is thus plausible that the impact of trait anxiety on connectivity may vary as a function of ongoing anxiogenic features within a movie.

By one view, impaired amygdala-prefrontal functioning in anxious populations might emerge through stable deficits in brain function (as tested within the resting-state framework). An alternate possibility is that idiosyncrasies in activity/functional connections change alongside the emotional content of movies [as evidenced in (18)]. Analogously, it has been demonstrated that increasing cognitive demands (via cognitive tasks) boosts brain-based predictions of cognitive variation compared with rest (19,20). Likewise, it may therefore be that individual differences in trait anxiety only surface within specific emotional contexts, most prominently state anxiety [as theorized by the diathesis-stress model (21) and as implicated by threat-of-shock studies (2)]. In this project, we aimed to extend our previous work on the engagement of threat circuitry during naturalistic viewing. Specifically, we aimed to explore the extent to which intersubject similarity in amygdala connectivity during movie watching was modulated as a function of trait anxiety. Moreover, we sought to test how anxiety-relevant differences in connectivity may vary as a function of the anxiogenic content within the movie (i.e., suspense).

### Hypotheses

We made the following preregistered (https://osf.io/hfc9n/) predictions in regard to a movie-watching fMRI dataset. Each were tested on left and right amygdala connectivity separately:

H1: Pairwise similarity in self-reported anxiety will positively correlate with similarity in amygdala-dmPFC connectivity during movie watching. In other words, we will observe anxiety-relevant idiosyncrasies when comparing subjects’ amygdala-prefrontal connectivity time courses across an entire movie clip.

H2: Pairwise similarity in self-reported anxiety will show greater correlations with amygdala–dorsomedial prefrontal similarity during highly suspenseful scenes. In other words, we will observe a greater impact of trait anxiety on amygdala-prefrontal connectivity during high (vs. low) suspenseful scenes.

## Methods and Materials

### Cambridge Centre for Ageing Neuroscience Dataset

#### fMRI Data

We conducted analyses on the Cambridge Centre for Ageing Neuroscience database (CamCAN) [*n* = 652, mean age = 54.81, SD = 18.54, 329 female, 50/589 left-/right-handed, 11 ambidextrous, 2 missing hand data (22,23)]. Participants were required to be cognitively healthy and free of neurologic or serious psychiatric conditions. Experimental procedures relevant to this study included viewing a clip from Alfred Hitchcock’s *Bang You’re Dead*. Blood oxygen level–dependent signal was acquired with multi-echo T2∗ echo planar imaging (32 axial slices 3.7-mm thick, 0.74-mm gap, repetition time [TR] = 2470 ms; echo time = 9.4, 21.2, 33, 45, and 57 ms; flip angle = 78°; field of view = 192 × 192 mm; 3 × 3 × 4.44 mm; acquisition time = 8 min 13 seconds). Functional data were preprocessed using realignment and unwarping with fieldmaps, slice-time correction, transformation to Montreal Neurological Institute space, and despiking using outlying wavelet coefficients (no smoothing). For a full overview of database details, see (23).

#### Self-report/Behavioral Data

**Figure 1:**
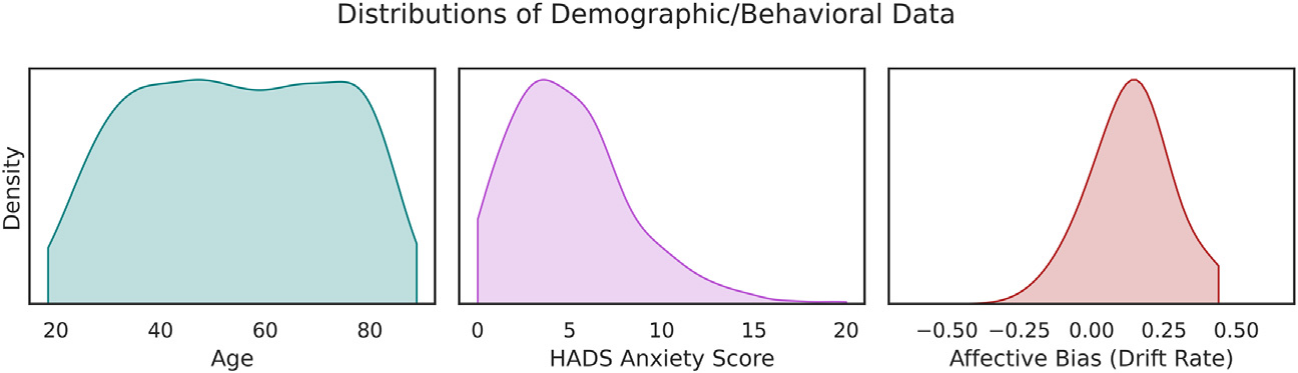
Kernel density plots for age, self-reported anxiety, and affective bias (cut at minimum/maximum). Due to a very low accuracy (5%, *z* = −10) in the face perception task, 5 subjects had a drift rate parameter of −3.1, which is not visualized here (but was retained in analyses). HADS, Hospital Anxiety and Depression Scale.

Prior to scanning, participants completed the Hospital Anxiety and Depression Scale (HADS) (24). The anxiety section of this scale constituted our self-report metric for hypothesis testing (Figure 1). Subjects with no available HADS data (*n* = 3) were omitted from the relevant analyses. In addition to self-report measures, we made use of canonical suspense ratings previously collected as 21 subjects viewed the same *Bang You’re Dead* clip (25). To account for hemodynamic lag, we shifted the ratings backward by 2 TRs [∼5 seconds; consistent with prior work in this domain (14)], removing the last two data points and imputing the first two with the mean of the first 5 TRs of the original ratings. This regressor therefore acted as a continuous, block-wide parametric modulator and did not require convolution with the hemodynamic response function.

In a set of exploratory analyses, we derived an anxiety-relevant cognitive bias measure from behavioral data. We were interested in whether individual differences in affective bias (26), namely greater vigilance toward threat-relevant stimuli in the environment (27), also demonstrated effects of movie-dependent connectivity [as observed in threat-of-shock studies (2)]. For this, we calculated affective bias measures from the face perception task participants completed prior to scanning (emotion expression recognition). This included labeling faces morphed between emotional expressions [happiness-surprise, surprise-fear, fear-sadness, sadness-disgust, disgust-anger, anger-happiness; stimuli derived from (28)]. Our affective bias measure was calculated through a simplified drift-diffusion model. We extracted summary statistics pertaining to accuracy and mean/variance of reaction time for correctly labeled trials where morphs contained 70%/90% fear (summary statistics used as trial-by-trial data are not provided within CamCAN). Summary statistics were then inputted into E-Z drift-diffusion modeling (29). The drift parameter constituted our affective bias metric. Reaction time variance values of 0 (one correct trial) and accuracy values of 0, 0.5, and 1 were increased (or decreased for the latter) by 0.000001 to avoid division errors. Subjects with no available face data (*n* = 15) were omitted from relevant analyses. Spearman correlation suggested a small, positive relationship between self-reported anxiety and affective bias (ρ = 0.13, *p* = .0008).

Our choice for the use of fearful (vs. angry) faces was based on a theoretical distinction between immediate, direct threat (an angry individual) versus uncertain threat (fearful expressions, which may signal the presence of nearby danger). The former could be conceptualized as evoking panic/fearful responses, whereas the latter may be more related to states of anxiety [although both likely fall on a continuum (30)].

### Analysis

fMRI time series extraction and modeling were conducted using AFNI (31) and Python. Relevant functions are denoted in parentheses. Analyses were preregistered (https://osf.io/hfc9n/) and used two-sided tests thresholded at α = 0.05. Post hoc analyses are reported separately in the results section. Visualizations were generated with Seaborn (32) and Matplotlib (33). Data can be accessed via a request to CamCAN (https://camcan-archive.mrc-cbu.cam.ac.uk/dataaccess/). We have made our scripts openly available (https://osf.io/5xsp6/).

#### Region of Interest Masks

**Figure 2:**
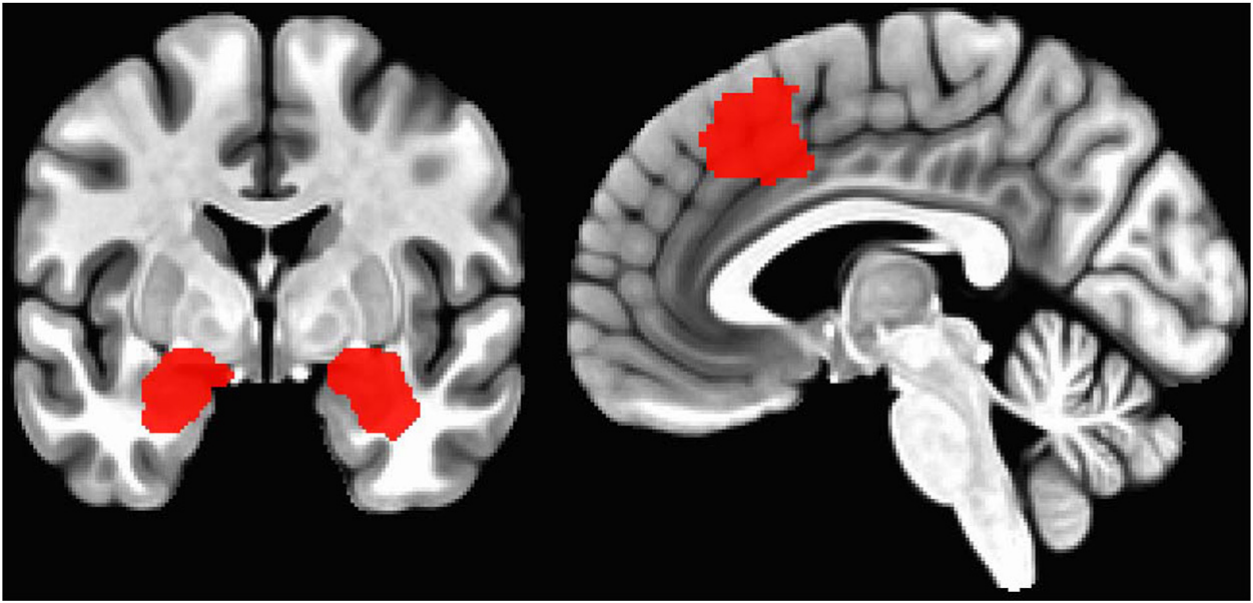
Region of interest definitions for hypothesis testing. Left: Subject-specific regions of interest were defined using FreeSurfer before being constrained within an inflated Montreal Neurological Institute amygdala mask (coronal slice at y = −2). Right: Our functional dorsomedial prefrontal cortex mask was generated from a meta-analysis looking at the conjunction between adaptive and maladaptive anxiety [sagittal slice at x = −3; Induced (+) vs. Transdiagnostic (+) 20 mm (36)].

Our amygdala regions of interest were selected through individual anatomical parcellations of T1 images in FreeSurfer (34) constrained with an inflated (3dROIMaker, -inflate 3) (Figure 1) Montreal Neurological Institute amygdala mask [Automated Anatomical Labeling atlas (35)]. The dmPFC was defined via a functional mask from a previous meta-analysis of anxiety-relevant task-based activations; specifically, we used a conjunction map of adaptive/maladaptive anxiety [Induced vs. Transdiagnostic 20 mm, cluster at ∼(0, 23, 45) https://neurovault.org/images/384691/ (36)] (Figure 2). For whole-brain analyses, functional volumes were segmented via a canonical parcellation (400 parcels) (37) constrained within subject-specific, inflated gray matter masks. Participants with failed FreeSurfer segmentations (*n* = 10) or no overlap between automasked echo planar imaging and at least one canonical parcel (*n* = 9) were excluded from analyses. Combined with missing self-report/behavioral data, this left 630 participants for our analyses on self-report measures (3.37% dropout) and 618 participants for analyses on affective bias measures (5.21% dropout).

#### Within-Subject/Pairwise Modeling: fMRI Data

We first removed effects of no interest from raw time series (3dDeconvolve) by regressing out baseline signals with drift (-polort A; demeaning/detrending) and 24 motion parameters (raw + derivatives + squares) to produce a cleaned time series for each voxel. We then extracted region of interest seeds (3dmaskave) from the cleaned volumes. These were taken forward to produce TR-wise functional connectivity measures based on sliding window analyses [timecorr: width = 8 TRs/∼20 seconds, gaussian kernel weighting (38)] between the amygdala and cortical regions of interest (which were subsequently Fisher transformed and *z*-scored).

Movie-wide intersubject representational similarity matrices were then constructed as a function of between-subject Pearson correlations in amygdala (left and right separately)-cortical connectivity measures. TR-wise intersubject representational similarity matrices were constructed as a function of differences in between-subject amygdala-dmPFC connectivity measures.

#### Within-Subject/Pairwise Modeling: Self-report and Behavioral Data

Self-report similarity measures were first calculated as the difference in self-reported anxiety. This allowed us to test a one-to-one relationship between anxiety and connectivity; namely, whether high-high or low-low anxiety pairwise comparisons showed greater similarity than high-low anxiety comparisons. In other words, participants who differ on the low end of the HADS scale (e.g., 1 vs. 2) will show the same differences in connectivity than those who differ on the higher end (e.g., 19 vs. 20). In instances of undirected brain measures (i.e., movie-wide correlations) we used absolute differences in self-reported anxiety.

We also generated an exploratory matrix using the AnnaK approach (19). Each cell in this matrix was calculated as the pairwise means of self-reported anxiety scores. Unlike the previous matrix, this allowed us to test a nonlinear relationship, namely that high-high anxiety pairwise comparisons would show greater similarity than low-low comparisons (or vice versa). For instance, a negative correlation would suggest that participants who differ on the higher end of the HADS scale (e.g., scores of 19 vs. 20) will show similar connectivity profiles, whereas participants who differ on the low end of the scale (e.g., scores of 1 vs. 2) show greater variability in connectivity profiles. Finally, we created a matrix for our affective bias measures, using both differences and AnnaK mean scores.

#### Group Modeling

**Figure 3:**
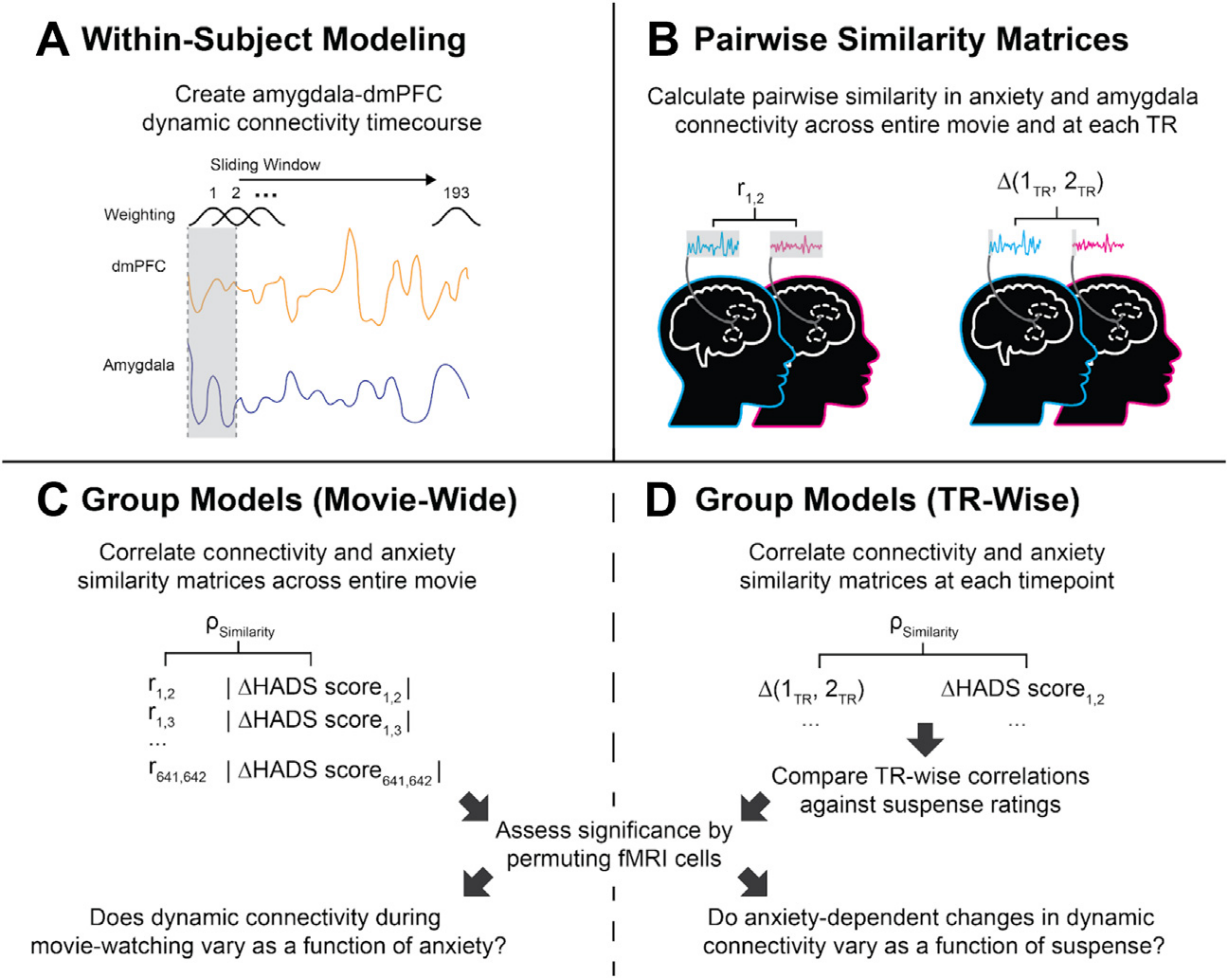
Illustration of analysis pipeline. **(A)** We first derived time series of dynamic connectivity using a sliding window approach, with data points in the window weighted using a Gaussian function. **(B)** Pairwise similarity matrices were produced by correlating dynamic connectivity time series, calculating differences in connectivity at each repetition time (TR), and calculating differences in anxiety measures. **(C)** To test our first hypothesis, we correlated pairwise similarities in anxiety and amygdala–dorsomedial prefrontal cortex (dmPFC) connectivity across the entire movie. **(D)** Testing our second hypothesis, we repeated this procedure but at every TR in the movie and compared anxiety-related differences in connectivity against canonical suspense ratings. Significance for all group-level models were based on permutation testing. fMRI, functional magnetic resonance imaging; HADS, Hospital Anxiety and Depression Scale.

We compared neural and behavioral similarity matrices using Partial Spearman Rank correlations with age, gender, and motion (mean framewise displacement) as covariates. TR-wise analyses against suspense ratings used Pearson correlations. Significance was based on null distributions derived from 10,000 permutations of cells in the neural similarity matrices. For an overview of our analysis pipeline, see Figure 3.

## Results

### Movie-wide Connectivity Tests

First, we conducted Spearman correlations between self-report similarity (absolute difference) and functional connectivity similarity across the entire movie clip (hypothesis 1; representational similarity matrices visualized in the Supplement). We did not observe effects in either left (ρ = 0.002, *p* = .297) or right (ρ = 0.001, *p* = .702) amygdala-dmPFC connectivity. Planned exploratory analyses also failed to show this for our affective bias (left: ρ = −0.003, *p* = .189; right: ρ = −0.003, *p* = .181) or AnnaK (left: ρ = 0.002, *p* = .362; right: ρ = −0.0002, *p* = .948) models. In further planned exploratory analyses, we reconducted movie-wide tests (self-report) using the Schaefer 400 cortical parcellations (37) for both absolute difference and AnnaK models. Although some parcels surpassed Bonferroni correction (400 parcels, *p* < .000125), effect sizes were marginal (max |ρ| = 0.03). In other words, when comparing connectivity across the entirety of the movie clip, no single amygdala parcel time series explained >0.09% of the variance associated with anxiety.

### Anxiety × Connectivity × Suspense Tests

**Figure 4:**
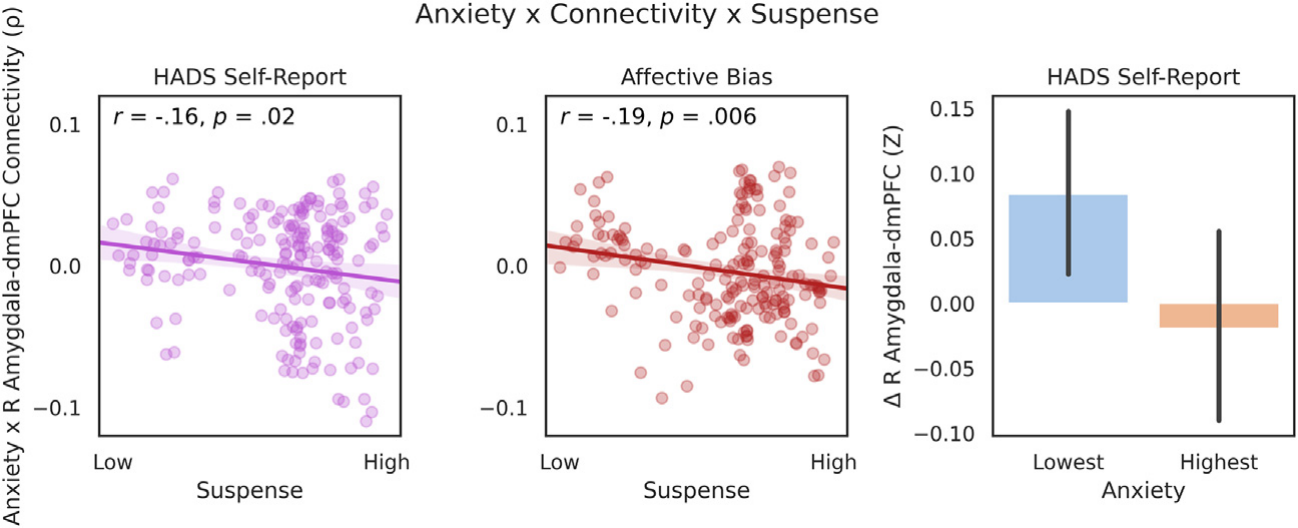
Left/Middle: Scatterplots demonstrating a negative correlation (with 95% confidence intervals) between repetition timewise suspense ratings and anxiety-relevant increases in right (R) amygdala–dorsomedial prefrontal cortex (dmPFC) connectivity. Left: Hospital Anxiety and Depression Scale (HADS) anxiety scores. Middle: affective bias (drift rate from drift-diffusion models of fearful face responding). Right: bar plot demonstrating change in average connectivity (*z* scores) from low to high suspense (highest − lowest quartiles of suspense) across the lowest and highest quartiles of self-reported anxiety (with 95% confidence intervals).

We next produced Spearman correlations between self-reported anxiety (constant) and neural similarity matrices for each TR (dynamic). TR-wise coefficients were then taken forward to Pearson correlations against the canonical suspense ratings time series. This allowed us to test whether mapping between amygdala connectivity and self-report similarity was most prominent during high suspense scenes (hypothesis 2). Unlike our movie-wide analyses, the TR-wise representational similarity matrices were directional in nature, meaning relative connectivity strength could be compared across subjects. We observed an inverse relationship to the one that we predicted: there was a significant negative correlation between canonical suspense ratings and anxiety-dependent increases in right amygdala–dmPFC connectivity (*r* = −0.16, *p* = .02), although this was not apparent for the left amygdala (*r* = −0.05, *p* = .53). Moreover, planned exploratory analyses demonstrated a stronger relationship between suspense and the impact of affective bias on right amygdala–dmPFC connectivity (*r* = −0.19, *p* = .006; left amygdala–dmPFC: *r* = −0.05, *p* = .51) (Figure 4).

**Figure 5:**
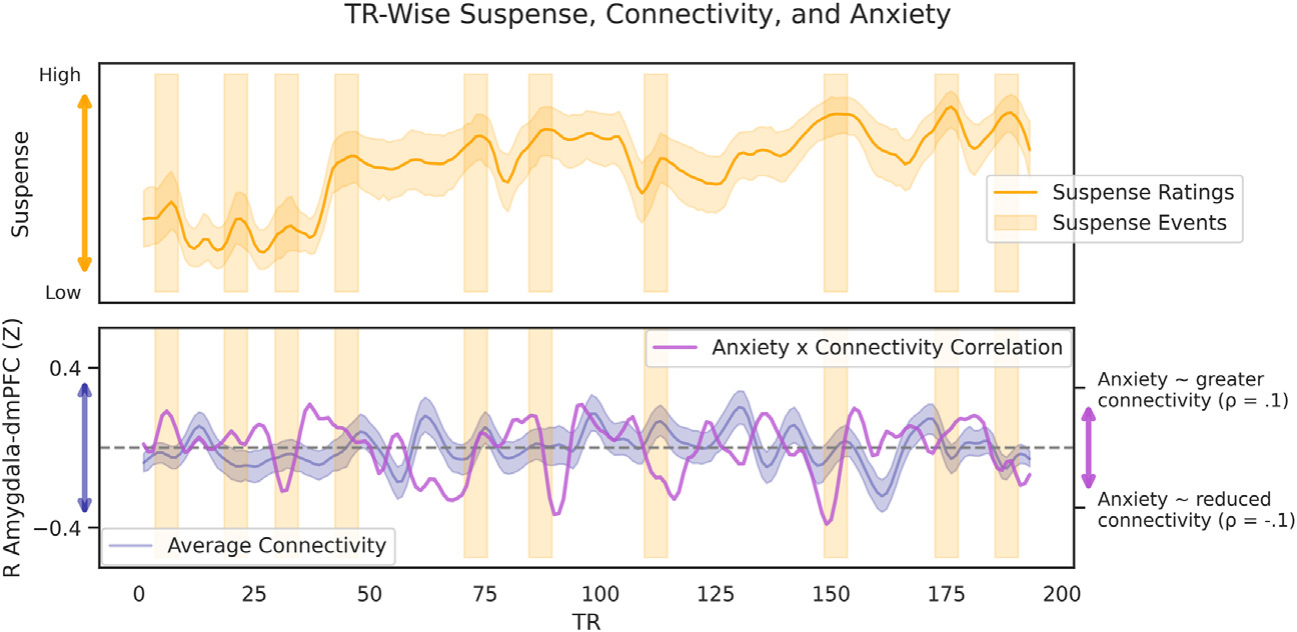
Time series of canonical suspense ratings (orange line), suspenseful events (orange rectangles, marked using amplitude-based peak detection), average right (R) amygdala–dorsomedial prefrontal cortex (dmPFC) dynamic connectivity, and the correlation between anxiety and dynamic connectivity at each repetition time (TR) (smoothed). Shading denotes 95% confidence intervals.

These results suggested that amygdala-dmPFC circuitry was modulated by suspense in low-anxiety individuals, but this circuitry was less sensitive to suspense in high-anxiety individuals. However, there are several interpretations for this result (see Discussion). To aid in interpretation, we ran post hoc amplitude-based peak detection [SciPy’s find_peaks (39)] across the suspense ratings time series (smoothed) to mark events of relative increases in suspense; this allowed us to visualize how anxiety-relevant alterations in amygdala-dmPFC connectivity altered alongside anxiogenic scenes (Figure 5). One event (#8) was manually adjusted to better reflect the plateau of suspense.

### Post Hoc Tests

Because the relationship between dynamic connectivity and anxiety appeared dependent on the presence of suspense, we reconducted TR-wise tests across a wider “defensive response network” (40) consisting of the amygdala, bed nucleus of the stria terminalis, hypothalamus, periaqueductal gray, subgenual anterior cingulate cortex, an anterior section of the ventromedial PFC, dmPFC, and anterior insula. Most prominently, suspense showed the strongest relationship with anxiety-relevant differences in amygdala–periaqueductal gray connectivity (left amygdala: *r* = −0.41, *p* < .0001; right amygdala: *r* = −0.35, *p* < .0001) (see the Supplement).

Working with unconstrained naturalistic data (i.e., movies), it is difficult to orthogonalize features across the stimulus. As such, results should be interpreted with an understanding that a degree of collinearity likely exists between low- and high-level stimulus features. Using the pliers package (41), we extracted features to demonstrate such collinearity. For instance, suspense showed small to moderate correlations with power of the audio signal (loudness: *r* = 0.41), brightness (*r* = 0.23), and number of faces present (*r* = −0.25) (Supplement). We also demonstrated correlations between age and anxiety (self-report: ρ = −0.23, *p* < .001; affective bias: ρ = −0.47, *p* < .001). Thus, there may be interactions between age and anxiety-dependent connectivity (for an analysis, see the Supplement).

We used a sliding window approach due to its utility demonstrated from previous work (42, 43, 44). However, a possible limitation of a sliding window approach to dynamic connectivity is that results may be sensitive to window length, offset, and filtering (45). As such, we tested the robustness of these effects when using differing window lengths. Using window lengths of 6 TRs (∼15 seconds), 8 TRs (∼20 seconds), or 10 TRs (∼25 seconds) did not change inference regarding suspense, right amygdala–dmPFC connectivity, and self-reported anxiety (6 TRs: *r* = −0.17, *p* = .02; 10 TRs: *r* = −0.17, *p* = .02). The same was true for our affective bias measures (6 TRs: *r* = −0.18, *p* = .01; 10 TRs: *r* = −0.20, *p* = .006). These analyses suggest that our reported findings are robust across a variety of window lengths.

## Discussion

There is a wealth of task-based literature implicating an amygdala-prefrontal circuit that underlies threat-relevant biases fundamental to anxiety (1, 2, 3, 4, 5,9). Yet, little has been done to test whether individual differences in this circuit arise in more naturalistic settings. In this study, we aimed to extend this work to a movie-watching paradigm using a dynamic, flexible analytic framework, intersubject representational similarity analysis. Here, we tested whether anxiety would correlate with amygdala-dmPFC dynamic connectivity throughout movie watching. We failed to find evidence for this. We next tested the hypothesis that the relationship between anxiety and connectivity would vary depending on anxiogenic features within the movie (i.e., canonical suspense ratings). We observed effects in the inverse direction to what we predicted: the relationship between anxiety and right amygdala–prefrontal connectivity was negatively correlated with suspense (*r* = −0.16, *p* = .02). In addition, a planned exploratory analysis suggested that a measure of affective bias (i.e., accuracy/reaction time to fearful faces) was slightly more sensitive to these effects (*r* = −0.19, *p* = .006). We offer several interpretations for how this effect may have arisen.

At face value, the negative relationship suggested that high (vs. low) anxiety individuals had relatively increased connectivity during low suspense scenes (and/or reduced connectivity during high suspense). This would suggest that high-anxiety individuals chronically engage this circuit, irrespective of anxiogenic scenes in movies, whereas low-anxiety individuals selectively engage this circuit in response to anxiogenic scenes. Some resting-state studies have evidenced greater sustained engagement of this circuit irrespective of stimuli (46), although there is mixed evidence (47). However, we suggest that this is not the most plausible inference. In a separate study of CamCAN resting-state data using the same subjects, masks, and self-report/behavioral measures, we did not find evidence for differences in intrinsic functional connectivity of amygdala-dmPFC circuitry (48). Moreover, this interpretation is in direct contrast to findings from threat-of-shock studies, which suggests that individual differences emerge primarily when under a state of anxiety (2).

One possibility is that anxiogenic, vicarious features of the movie evoke different affective processes to those elicited by direct, personal threat (threat of shock). There is scant evidence explicitly investigating how the medium of anxiety induction impacts brain response. Behavioral research has suggested differential impacts of physical versus social threats on emotional face perception among socially anxious individuals (49). In addition, given the very distinction between social and generalized anxiety disorders (50), social versus direct threats may indeed differentially affect anxiety-relevant processes. Self-reported findings from media psychology offer additional insight. Unlike threat of shock, which is not typically thought of as a desirable experience, many people seek out anxiogenic media such as horror movies (51). Indeed, it has been suggested that while the initial experience of anxiogenic scenes may be aversive, individuals scoring high in sensation seeking may feel an aftermath of positive emotions, and emotionally unstable individuals may show greater evoked anxiety (52). Moreover, viewers of anxiogenic media may be worried about characters within the movie but not themselves. Given that our anxiety measure was self-oriented, the suspense ratings may also be affected by trait empathy. Therefore, the affective state elicited by suspenseful movies is likely multifaceted and more dynamic in nature than states evoked by threat of shock. Given the absence of these effects while at rest (48), we conclude that these individual differences are likely arising in response to the emotional context elicited by the movie.

Visualization of our results further supports a distinct interpretation to suspense-insensitive, chronic engagement. Although temporal fluctuations in suspense resulted in initially similar right amygdala–dmPFC connectivity patterns between participants, toward the end of and/or following a lag after these events, we see a divergence in coupling as a function of individual differences in anxiety. This is in line with a body of literature demonstrating that anxious individuals have reduced habituation to threat-relevant stimuli (53, 54, 55). This also relates to findings demonstrating an association between personality and emotions experienced after anxiogenic scenes (52). In other words, we suggest that engagement of amygdala-prefrontal connectivity was slower to taper off following anxiogenic scenes in high-anxiety individuals. Inversely, due to the short intervals between suspenseful scenes, this could be explained by amplified effects of anxious anticipation [engagement of amygdala-prefrontal connectivity was stronger when high-anxiety individuals started to expect a forthcoming suspenseful scene (56,57)].

We did not submit these time series to any formal analyses because this would have relied on post hoc assumptions regarding data that we had already observed, such as the specific lag following suspense events. Moreover, the aforementioned delay appears nonconstant and/or could be affected by other features not modeled in this study (which was limited to canonical suspense ratings). Thus, the explanations we offer are of course provisional. Nonetheless, our results provide evidence that anxiety, dynamic connectivity, and anxiogenic features of a movie do interact in a time-varying manner. An opportunity movie-watching fMRI offers for future research is elucidating exactly how anxiety, connectivity, and nested features of the stimuli may interact. Based on these findings, we encourage future research to explicitly test temporal lags in modeling (e.g., vector autoregression); embed various features of the stimulus, ranging from high-level affective dynamics (e.g., suspense) to visual onsets (e.g., facial expressions), in analyses of dynamic connectivity; and compare connectivity profiles between anxiogenic scenes and threat of shock.

We also draw attention to the convergence between our self-report [HADS (24)] and behavioral measures (affective bias, derived from accuracy and reaction times to fearful facial expressions). The correlation between self-report and behavior was small (ρ = 0.13, *p* = .0008), suggesting that—although overlapping—these measures could tap into different latent constructs. Whereas the latter targeted attentional biases to threat, a key feature of anxiety (26,27), the self-report measure summated multiple symptoms of pathological anxiety (e.g., “Worrying thoughts go through my mind,” “I get a sort of frightened feeling like ‘butterflies’ in the stomach”), which may measure distinct dimensions [e.g., worry, somatic symptoms, interoception (58,59)]. We were unable to assess item-level correlations in this dataset. Nonetheless, both self-report and affective bias demonstrated correlations with dynamic right amygdala–dmPFC connectivity and suspense. Yet, the relationship between affective bias, connectivity, and anxiogenic features of the movie (*r* = −0.19, *p* = .006) was slightly stronger than self-reported anxiety (*r* = −0.16, *p* = .02). These results lend support for previous theorizations that this circuit is involved in attentional amplification of threat-relevant stimuli, such as emotional facial expressions (9); this may also be related to how our affective bias measure was based on a perception of others and the stimulus presented posed danger to other characters.

We note several facets of these findings that may warrant further investigation. First, we highlight the relationship between anxiety and right (but not left) amygdala–prefrontal connectivity. This lateralization is congruent with previous threat-of-shock studies (2,5,60), yet little is known about this dominance. Given that lateralization is apparent both within and outside of traditional paradigms, this warrants further investigation (e.g., whether this is related to handedness). Second, we note that there may be potential interactions between age, anxiety, connectivity, and suspense (Supplement). Future work should seek to detail the exact nature of this relationship. Third, we were unable to test whether the observed associations are apparent in those with a clinical diagnosis. There is evidence to suggest that the impact of induced anxiety may vary as a function of clinical diagnosis (61). Therefore, it is possible that these effects may not manifest in the same manner for those with clinically significant levels of anxiety, and results need to be interpreted in the context of subclinical variation. To our knowledge, there are currently no available movie-watching datasets that have explicitly sought to test clinically diagnosed individuals. This may prove fruitful for further exploration of the impact of anxiety on brain responses to movie watching.

Finally, we highlight the unconstrained nature of this paradigm. Given the naturalistic basis of the stimulus (a movie), it is unsurprising that there are confounded features within the stimuli. We noted small to moderate correlations between suspense ratings, power of the audio signal (loudness), brightness, and faces present. However, these aesthetics likely culminate to give rise to overall suspense (62). It is therefore difficult to elucidate sensory processing from affective phenomena. Given that the relationship between anxiety and connectivity manifested in a slightly different manner to that seen in threat-of-shock studies, it will be important to demonstrate generalizability of this effect across different movie stimuli, preferably with less collinearity between features, and ensure that future task-based and movie fMRI studies are conducted in compliment to each other.

### Conclusions

In this study, we investigated whether dynamic connectivity during movie watching related to individual differences in anxiety. Across the entirety of the movie clip, comparisons of dynamic amygdala-prefrontal connectivity did not relate to individual differences in anxiety. However, anxiety appeared to have a variable impact on dynamic connectivity dependent on the presence of anxiogenic features in the movie (i.e., suspense). We suggest that anxiety could be associated with suspense-insensitive, chronic engagement of threat circuitry in high-anxiety individuals; slowed habituation of threat circuitry following anxiogenic scenes; or greater apprehension of anxiogenic scenes. Elucidating exactly how and when these individual differences appear offers opportunity for future study.

## Supplementary Material

Supplementary Material

Key Resources Table
