# Erratum

**DOI:** 10.1016/j.bpsgos.2023.05.002

**Published:** 2023-05-24

Erratum to: “Anxiety Shapes Amygdala-Prefrontal Dynamics During Movie Watching,” by Kirk *et al.* (*Biol Psychiatry Glob Open Sci* 2023; 3:409–417); https://doi.org/10.1016/j.bpsgos.2022.03.009.

The authors discovered an error in their code for deriving *p* values via permutation testing. Specifically, a change in the multi-threading factor resulted in fewer permutations generated for the null distribution than had been preregistered, which were then divided incorrectly. This affected only the movie-wide tests (not associations with suspense). The authors have corrected this error and rerun their analyses, which did not change statistical inference.

As a result of this error, the 6 reported *p* values in the Movie-wide Connectivity Tests section of the Results required updating. These *p* values all remained nonsignificant, leaving the overall results and interpretations unaffected.

The *p* values have been corrected in the final paginated version of this article.

