# Supplementary material for: Anxiety Shapes Amygdala-Prefrontal Dynamics During Movie Watching
Source: Biol Psychiatry Glob Open Sci. 2022 Mar 31;3(3):409–17. doi: 10.1016/j.bpsgos.2022.03.009 (PMC10382705; doi:10.1016/j.bpsgos.2022.03.009)
Supplement: Supplementary Material [file mmc1.pdf]

Supplementary Information

Anxiety Shapes Amygdala-Prefrontal Dynamics During Movie-Watching

Representational similarity matrices

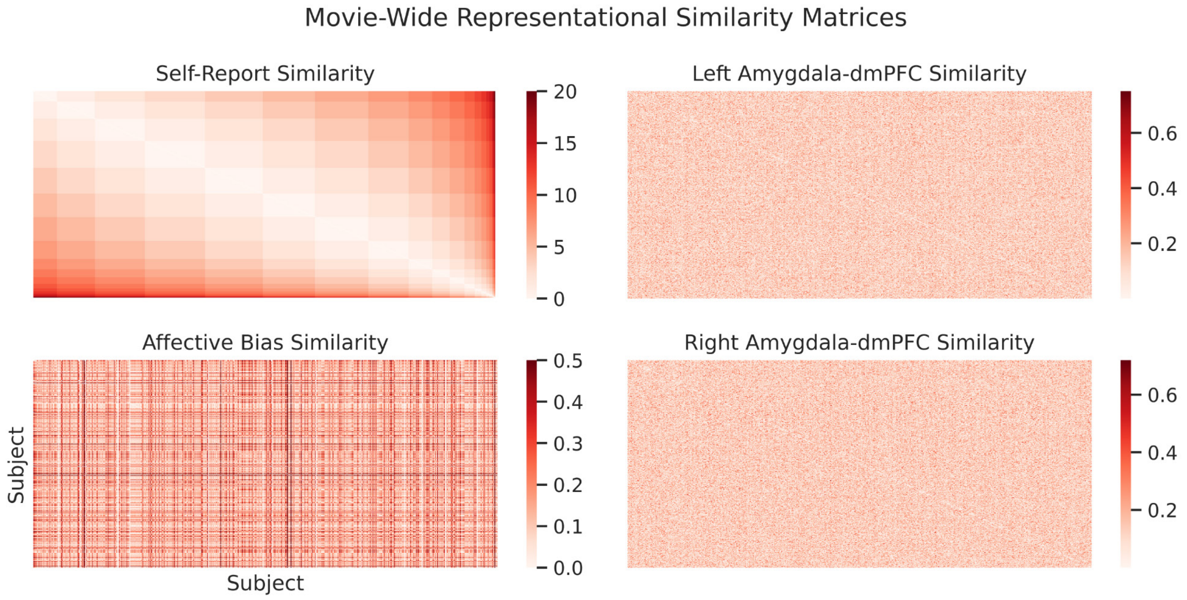

Supplemental figure 1. Representational similarity matrices for self-reported anxiety, affective bias, movie-wide left amygdala-dmPFC similarity, and movie-wide right amygdala-dmPFC similarity. Subject rows and columns sorted in ascending order of self-reported anxiety.

Effects across the ‘defensive response network’

As the relationship between dynamic connectivity and anxiety appeared dependent on the presence of suspense, we reconducted TR-wise tests across a wider ‘defensive response network’ (1) consisting of amygdala, bed nucleus of the stria terminalis, hypothalamus, periaqueductal gray, subgenual anterior cingulate cortex, an anterior section of the ventromedial prefrontal cortex, dorsomedial prefrontal cortex, and anterior insula (for details regarding mask definition, see (2)).

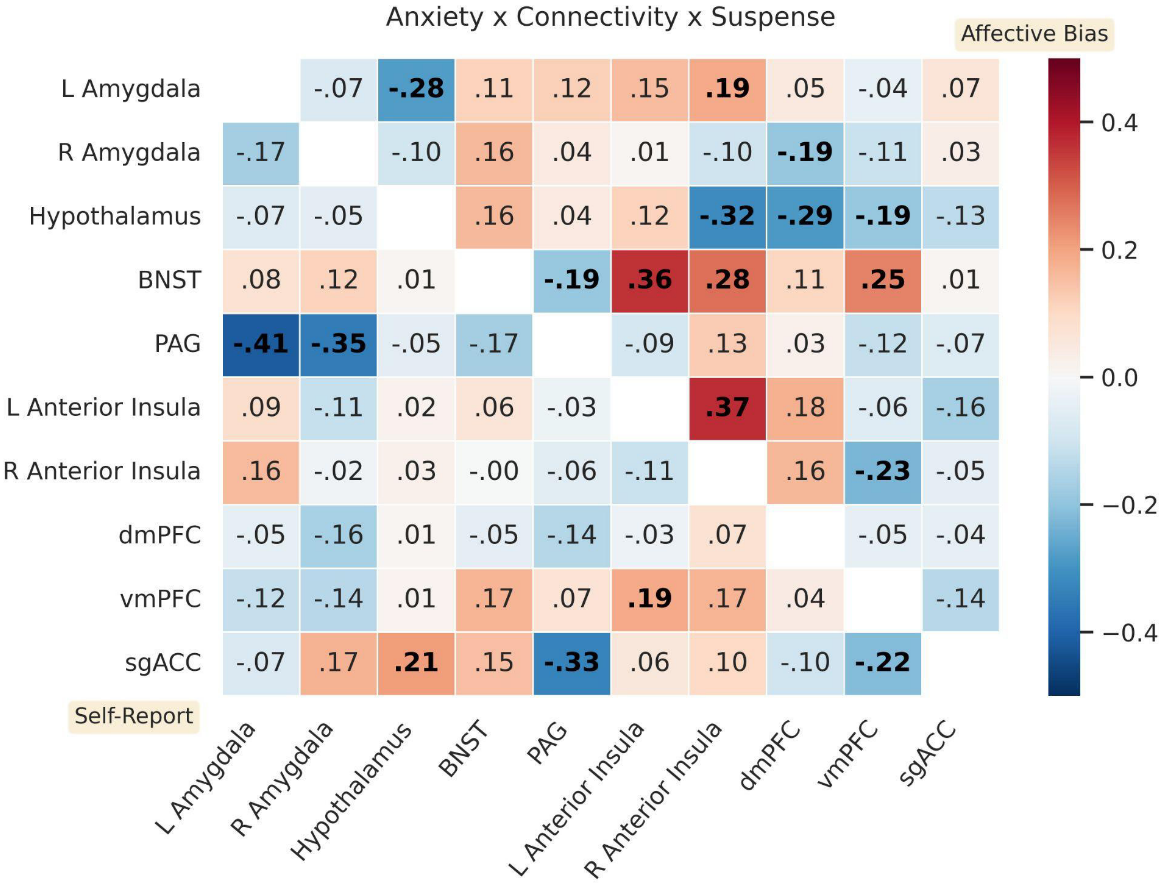

Supplemental Figure 2. Pearson correlations between TR-wise suspense ratings and anxiety-relevant increases in dynamic connectivity (lower triangle = self-report; upper triangle = affective bias). Bolded cells refer to correlations surviving Bonferroni correction across 45 ROI x ROI comparisons, $p < .0011$.

Feature collinearity

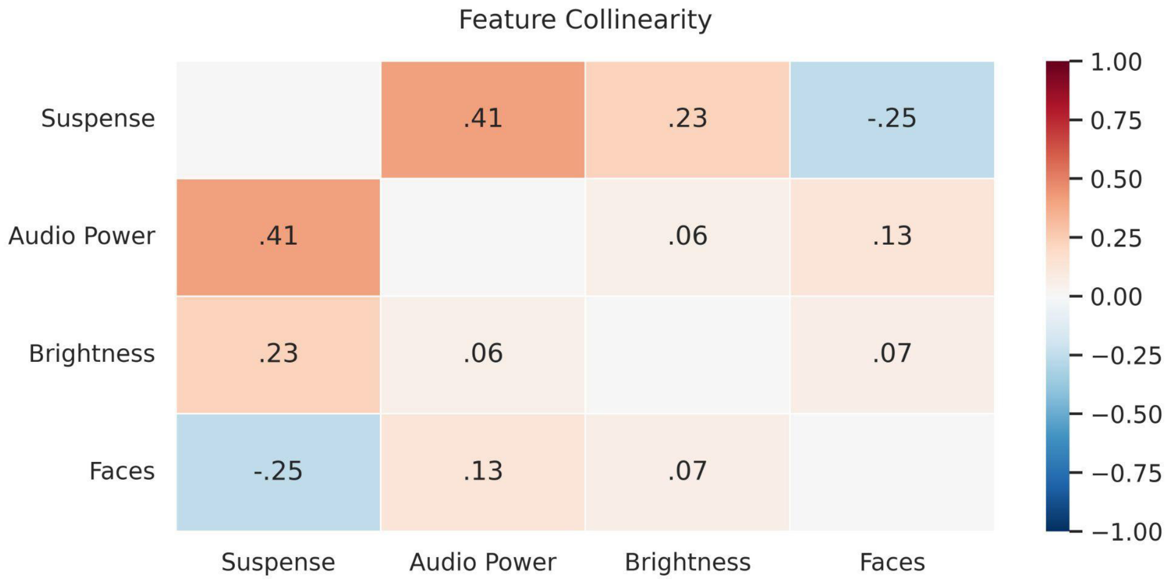

Supplemental Figure 3. Feature collinearity. Pearson correlation heatmap of features (canonical suspense, audio signal power, brightness, and number of faces present) present in the movie stimulus.

Effects across ages

To guide future applications of movie-watching data, we reconducted tests with a focus on age. We firstly note that there appears to be inherent associations between age and self-reported anxiety/affective bias (supplemental figure 4). Next, we discretized data according to age quantiles ([18.5 - 38.9], [38.9 - 54.3], [54.3 - 70.1], [70.1 - 88.9]) and re-ran anxiety x suspense tests. These tests indicate interactions are likely to exist between age, anxiety, dynamic connectivity, and suspense (supplemental table 4). Future work should seek to detail the exact nature of this relationship.

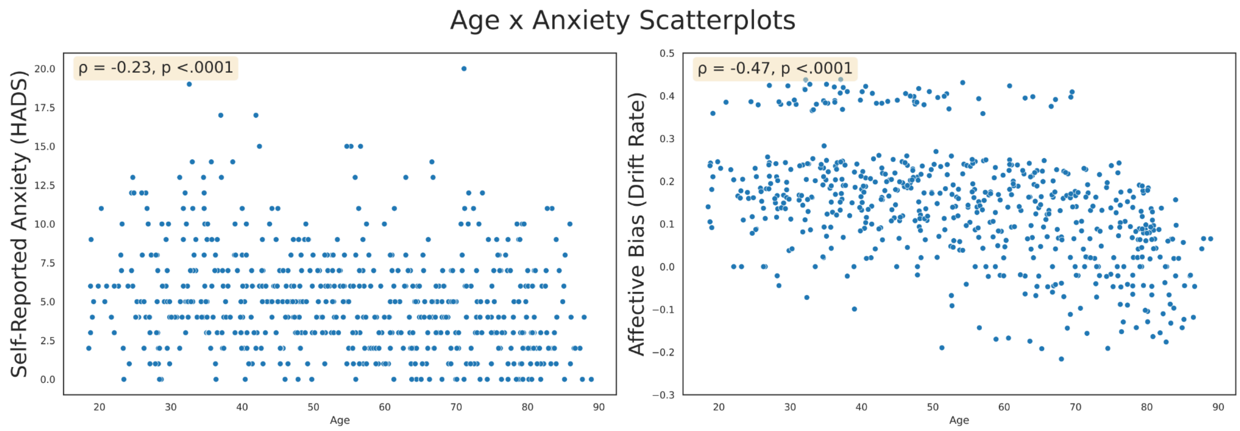

Supplemental figure 4. Scatterplots of self-reported anxiety (left) and affective bias (right) as a function of age. Note: due to a very low accuracy (5%, $Z = -10$) in the face perception task, five subjects had an affective bias score of (drift rate parameter) of -3.1 which is not visualized here (but was retained in analyses).

Supplemental table 4. Anxiety/Bias, connectivity, and suspense correlations across age quartiles

**Age (years)**	**Self-reported anxiety**		**Affective bias**	
	r	*p*	*r*	*p*
18.5 - 38.9	.12	.1	.03	.68
38.9 - 54.3	-.13	.07	-.16	.02
54.3 - 70.1	-.33	<.001	-.11	.11
70.1 - 88.9	.03	.62	-.23	.001

Supplemental References

1. Abend R, Ruiz SG, Bajaj MA, Harrewijn A, Linke JO, Atlas LY, *et al.* (2022): Threat imminence reveals links among unfolding of anticipatory physiological response, cortical-subcortical intrinsic functional connectivity, and anxiety. *Neurobiol Stress* 16: 100428.
2. Kirk PA, Holmes A, Robinson OJ (2022, January 11): Threat Vigilance and Intrinsic Amygdala Connectivity. PsyArXiv. https://doi.org/10.31234/osf.io/xjspq
